# The Role of Copper Intake in the Development and Management of Type 2 Diabetes: A Systematic Review

**DOI:** 10.3390/nu15071655

**Published:** 2023-03-29

**Authors:** Sereen Eljazzar, Haya Abu-Hijleh, Dana Alkhatib, Sara Sokary, Shrooq Ismail, Ghadir Fakhri Al-Jayyousi, Reema Tayyem

**Affiliations:** 1 Department of Human Nutrition, College of Health Sciences, QU Health, Qatar University, Doha P.O. Box 2713, Qatar; 2 Department of Public Health, College of Health Sciences, QU Health, Qatar University, Doha P.O. Box 2713, Qatar

**Keywords:** copper intake, type 2 diabetes, systematic review

## Abstract

Diabetes mellitus is a worldwide public health issue with numerous complications. Several risk factors are associated with diabetes, mainly due to patients following an unhealthy lifestyle. Copper is a crucial trace element, with various physiological actions. Different intake levels of copper might contribute to diabetes development due to its dual action as both an anti- and pro-oxidant. Aim: Due to the inconclusive findings regarding the relationship between copper consumption and the management of diabetes, we decided to conduct this extensive systematic review. Up to this date, no similar study has been available in the literature. In this review, we used the preferred reporting items for systematic reviews and meta-analysis (PRISMA) guidelines. Relevant articles were identified by searching the electronic databases CINAHL, EMBASE and Medline from their respective index dates to September 2022 using keywords such as “Copper Intake” and “Type 2 Diabetes”. Any paper that has investigated copper exposure through supplementation or any other method that indicates copper intake in human subjects with type 2 diabetes and measures at least one of the outcomes of interest related to diabetes was included in this review. This review is comprised of 4 cross-sectional studies, 3 cohort studies, 2 RCTs, and 2 interventional studies. Two cohort studies found positive associations between copper intake and the risk of developing T2DM, while no significant association was found in the third study. Regarding diabetes outcomes in the four cross-sectional studies, two found inverse associations, one found a positive relationship, and one found no significant association. In interventional studies, all studies found a protective effect of copper, including the RCT, while one found no significant association. The results are inconsistent concerning the association between copper consumption and the likelihood of developing diabetes are inconsistent. Individuals should receive an adequate dietary amount of copper that is within the RDA levels (900 µg/day) to avoid copper deficiency or toxicity. Further studies, especially RCTs, are strongly needed to enable researchers to elucidate more robust conclusions regarding this association.

## 1. Introduction

Diabetes mellitus (DM) is a chronic disease that arises from either partial or complete insulin deficiency [1]. Diabetes is becoming a worldwide public health issue due to its high prevalence [2]. In 2014, the World Health Organization (WHO) reported that diabetes affected 8.5% of all adults aged 18 and above, as per their own statistics [3]. Moreover, in 2019, approximately 1.5 million of total deaths were directly related to diabetes. Prevalence studies revealed that, in contrast to high-income countries, low- and middle-income countries exhibited a higher percentage [4].

Diabetes is a global health burden, with many health impacts. There are several complications that diabetic patients suffer from which adversely affect their quality of life, such as cardiovascular complications, peripheral neuropathy, nephropathy, retinopathy, mental diseases, hearing difficulties and oral conditions [5]. As per the Centers for Diseases Control and Prevention (CDC), diabetes symptoms are characterized by increased feelings of thirst (polydipsia), increased urination (nocturia), fatigue, blurry vision, skin dryness, delay in wound healing, concurrent infections and tingling limbs [6].

Diabetes can occur due to several reasons. The primary etiology for type 1 diabetes is auto-immune conditions, which leads to the destruction of the beta cells responsible for producing insulin in the pancreas [7]. However, for type 2 diabetes, the factors are mainly related to lifestyle behaviors and genetics [8]. Crucial modifiable factors that can lead to the onset of diabetes include excess body fat, physically inactivity and following an unhealthy eating pattern. Genetic mutations, hormonal diseases, pancreatic conditions, like pancreatitis, pancreatic cancer or trauma, as well as some medications, might also contribute to the development of diabetes development [9]. According to the CDC, choosing healthy food choices, being physically active and managing stress are scientifically proven guidelines for diabetes prevention and these are recommended through their national diabetes prevention program [10].

Over the past few decades, several studies were carried out to study the association between copper and diabetes. Copper is a crucial trace mineral needed for the efficient metabolism of many physiological reactions [11]. Dietary sources high in copper include organ meats and nuts [12]. Fruits and cereals may also be a good source of copper but will vary depending on the soil concentration of copper, which varies according to regions, pesticide use, and industrial copper emissions [12]. Zinc shares many of the dietary sources of copper, but it is also present at high concentrations in oysters, whole-grain cereals, and red meats [12]. Copper is an important cofactor needed for redox reactions [11]. The condition of copper overload and deficiency can both lead to adverse health outcomes. The effect of copper on oxidation can vary depending on the level of intake, as it can function as both an anti-oxidant and a pro-oxidant [13]. The anti-oxidant properties of copper are demonstrated through its being an essential component of copper/zinc superoxide dismutase (SOD) [14]. SOD enables free radical clearance in all body cells, hence defending against oxidative stress [15]. However, copper toxicity might induce insulin resistance by acting as a pro-oxidant [16]. Excessive copper promotes the generation of reactive oxygen species (ROS), which increases the oxidative stress and eventually leads to diabetes [17].

Findings concerning the relationship between copper intake and diabetes are inconclusive and scarce, as the available studies show inconsistent results. Thus, we aimed to conduct this comprehensive systematic review to elucidate the studies which examines the association between the copper intake and diabetes as up to this date, no similar study has been made available in the literature. Therefore, this systematic review will be the first to compile and review the available evidence regarding the association between dietary copper intake and diabetes so that clinicians can have access to guidance about the importance of copper supplementation in the amelioration and/or prevention of diabetes. This systematic review will support researchers who are aiming to find evidence regarding this association and will facilitate conducting research in this field. In addition, it will identify gaps in the literature that need to be tackled to establish a better understanding of the association between dietary copper intake and diabetes.

## 2. Methods

This systematic review adheres to the guidelines of preferred reporting items for systematic reviews and meta-analysis (PRISMA) (Table S1).

### 2.1. Literature Search

**Figure 1:**
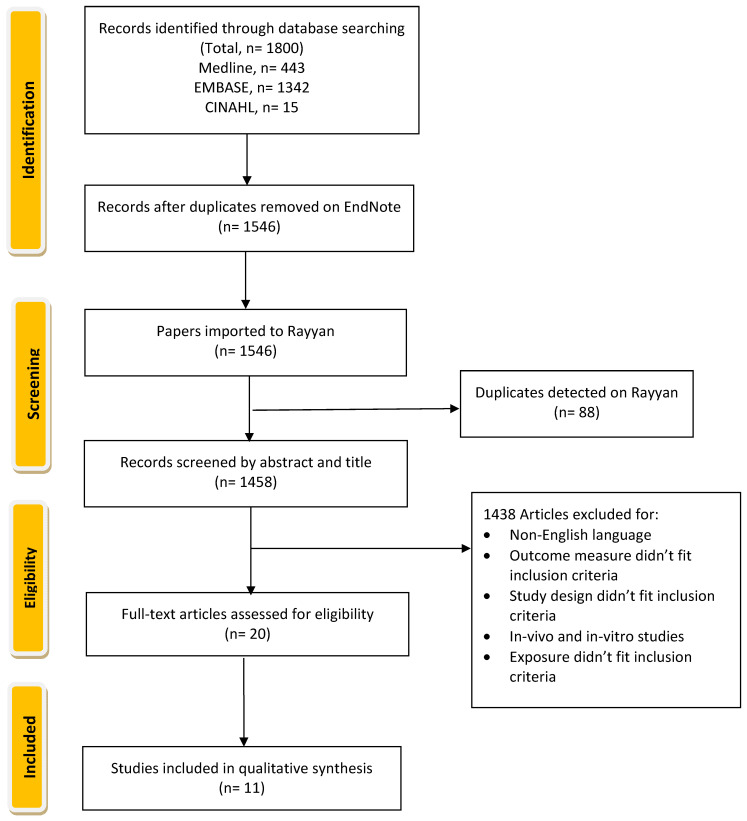
Flow chart of The Systematic Review of Copper Intake and Type 2 diabetes.

Articles of relevance were obtained by searching electronic databases Medline, EMBASE, and CINAHL from their respective index dates to September 2022. An additional free hand-search was conducted to ensure sure that this review includes almost all relevant papers and to reduce the possibility of missing any paper that could be included. The search strategy incorporated Medical Subject Headings, Emtree, and CINAHL subject heading (Table 1) for all three databases with search/key terms related to “Copper” and “Diabetes”. Articles from the three databases were imported into Endnote, where duplicates and articles composed in non-English languages were identified and removed. Articles were then imported into Rayyan Web for screening and assessment (Figure 1).

### 2.2. Study Selection

In our systematic review, studies were assessed for potential inclusion based on specific criteria which include any paper that has investigated copper exposure through supplementation or any other method that indicates copper intake in human subjects with type 2 diabetes and measures at least one of the outcomes of interest (glycated hemoglobin A1c (HbA_1c_), fasting plasma glucose (FPG), 2 h plasma glucose (2-H PG), oral glucose tolerance test (OGTT), blood lipids (high-density lipoprotein (HDL), low-density lipoprotein (LDL), triglycerides (TG), any diabetes complications and total cholesterol levels (total Chol)), as well as any diabetes-related complication. Articles that were not based on original research (commentaries, conference proceedings, letters, and reviews) were excluded, as were in vitro and in vivo studies. Five reviewers were involved to independently screen all the articles by publication titles and abstracts and then evaluate the full-text publications for inclusion in this review. Disagreements were resolved by the authors performing a second review through a consensus-based discussion.

### 2.3. Data Extraction

The authors extracted data using a standardized approach. For each article identified, we extracted information on the study characteristics including the first author’s family name, publication year, country, study design, settings, sample size, population, and sample characteristics (sex, age, baseline weight/health status); we additionally noted the copper administration, outcome assessment, and main findings of each study. Table 2 provides a summary of all articles included in this review.

### 2.4. Risk-of-Bias Assessment

We used the Newcastle–Ottawa scale (NOS) to conduct a quality assessment of the included studies [29]. This tool has three domains which assess selection, comparability, and outcome. For each study, a grade of a maximum of one point can be given for each item except comparability, which can be given a grade of up to 2 points. The evaluation of the studies’ quality is determined by their overall score on the Newcastle–Ottawa scale, which has a maximum score of 9. A rating of 0–2 suggests inadequate quality, 3–5 indicates moderate quality, while 6–9 implies good or high quality. To determine the quality of randomized controlled trials and other interventional studies, we employed the Cochrane risk-of-bias tool [30]. This tool evaluates the study across six categories: sequence generation, allocation concealment, blinding, incomplete outcome data, selective outcome reporting, and other potential threats to validity. Each domain is assessed with a ‘yes’, ‘no’, or ‘unclear’ response, indicating a low, high, or unclear risk of bias, respectively. If the first three questions receive a ‘yes’ response and no significant concerns are found in the last three domains, the study is classified as having a low risk of bias. If two domains receive an ‘unclear’ or ‘no’ response, the study is classified as having a moderate risk of bias. Finally, if three or more domains receive an ‘unclear’ or ‘no’ response, the study is classified as having a high risk of bias.

## 3. Results

### 3.1. Overall Quality Assessment

Descriptions of the main characteristics of the studies are presented in Table 2. A quality assessment was performed for all studies included in this systematic review. Based on the quality assessment of cross-sectional studies using the Newcastle–Ottawa scale [29], and as presented in Table 3, out of the four studies, only one study received a total quality score of 6, which indicates good quality. The other three studies received a total quality score of 5, which indicates studies to be of fair quality. This was mainly due to either issues with comparability or outcome assessments. Three cohort studies were included as well, and all were considered to be good or high-quality studies. To assess the RCT and other interventional trials involved in this review, we used the Cochrane risk-of-bias tool [30]. As presented in Table 4, out of the four trials included, only one had a low risk of bias. Two had an overall high risk of bias, and one had a serious overall risk of bias. The latter scenario was mostly due to being an old trial with a great deal of missing information.

### 3.2. Cross-Sectional Studies

Four cross-sectional studies were included in this systematic review. Zhang et al. used the NHANES database to conduct a cross-sectional study about the association between copper intake and the risk of diabetes nephropathy [20]. There were no significant differences between either group in term of hypertension or lipid profile. The study showed that as the intake of copper increases, the risk for diabetes nephropathy decreases, with a pooled odds ratio of 0.67 (95% CI: 0.54–0.84) [20]. A second cross-sectional study by Tan et al. into 128 overweight and obese Malaysian adults tested the association between dietary copper intake and insulin resistance [21]. All subjects had no diabetes, cardiovascular diseases, stroke, renal or endocrinal diseases. The study found that there is a noticeable and beneficial relationship between dietary copper and HOMA-IR, but only if the intake of copper is equal to or greater than 13.4 μg/kg/day, with an odds ratio of 0.276 (95%CI = 0.025–0.527, *p* value for trend = 0.033). Interestingly, the study found that the insulin-resistant group had significantly higher total cholesterol/HDL cholesterol and triglyceride and lower HDL cholesterol compared to the non-insulin-resistant group [21]. Another study by Norbitt et al. used the data from the ANDROMEDA project to assess dietary intake [22]. Subjects included in the study were diagnosed with AP between 2015–2019 with the most updated international diagnosis guideline and either did or did not use anti-diabetic medication or insulin use. The study excluded individuals with diabetes type 1 or gestational diabetes, chronic pancreatitis, intraoperative diagnosis of pancreatitis or post-endoscopic retrograde cholangiopancreatography pancreatitis. The results revealed a significant difference in the mean intake of copper between the 3 groups, as well as a significant negative association between the fasting plasma glucose and copper intake in the NAP group [22].

Sobhani et al. studied the association between the intake of fast food and multiple outcomes including anthropometric measurement, serum lipid profile and glucose metabolism in patients with diabetes nephropathy (DN) [23]. Participants were at stage 1 or 2 of diabetes, with a fasting blood glucose level of 126 mg/dL, the consumption hypoglycemic medication or insulin, and a proteinuria level of 300 mg/dL [23]. The results showed that a higher intake of fast food was associated with a higher intake of copper, among other nutrients [23]. In addition, it was found that copper intake was higher in those with higher intakes of fast food. This was related to higher levels of blood pressure and total cholesterol, rather than to any significant diabetic outcomes [23].

### 3.3. Cohort Studies

Three cohort studies were found to assess the impact of copper intake on diabetes parameters. The first one was performed by Eshak et al. aimed to assess the diet of the Japanese population [24]. After distributing food frequency questionnaires to their 16,160 subjects who have no risk of diabetes, cardiovascular diseases or cancer and following up with them for 5 years, 396 subjects developed T2DM (2.5%) [24]. This study showed that those who had higher dietary intakes of copper and iron had a higher risk of T2DM, with the risk being more pronounced among those who were older, overweight, smokers, and who had a family history of diabetes [24]. In the second cohort study, Cui et al. assessed the diet of 14,711 adults free of diabetes, with or without hypertension, and without any cardiovascular diseases in the China Health and Nutrition Survey and assessed the risk of those newly diagnosed with T2DM between 2000 and 2015 [25]. They found that the association between dietary copper and T2DM risk in healthy Chinese adults was not independent, but rather that copper was positively associated with a higher risk of developing T2DM, especially when selenium intake was lower than the medium (*p* interaction = 0.0292) [25]. The final cohort study by Laouali et al. monitored 70,991 women with or without hypertension or hypercholesteremia for 20 years, examining the association between T2DM incidence and dietary copper-to-zinc ratio [26]. After 20 years, they found that a copper-to-zinc ratio of less than 0.55 was associated with a lower risk of T2DM [26]. They also found that women with zinc intakes of less than 8 mg/day, a higher copper-to-zinc ration was associated with a higher T2DM risk, especially among obese females [26]. Higher copper-to-zinc ratios were associated with more frequent hypercholesterolemia and higher age and BMI at baseline [26].

### 3.4. Interventional Studies

Two RCTs and two interventional studies were performed to assess the impact of copper on diabetes outcomes. The randomized control trial by Gunasekara et al. aimed to evaluate the effect of a multimineral vitamin including copper, administered with or without zinc, on blood glucose levels in adult diabetics with or without essential hypertension and dyslipidemia. They excluded subjects with renal and liver disorders, those on hormonal therapy, those with a history of recent surgery, pregnant and lactating women and those who are undergoing insulin preparation therapy [18]. They that found that a combination of zinc and a multivitamin supplement (which had 2 mg of copper as part of its composition) reduced FBG by a mean of 0.33 mmol/L and reduced postprandial glucose levels by 1.55 mmol/L [18]. Therefore, they concluded that a multivitamin in addition to zinc could have beneficial effects in the management of diabetes in adults [18]. Alfawaz et al. also conducted a randomized control trial to assess dietary pattern changes in adults with prediabetes with no other chronic disease [19]. They observed copper deficiency at baseline for both groups but observed improved intakes of copper in the guidance group compared to the GA group (37.3% vs. 13.3%) as well as improvements in the fasting glucose levels and insulin resistance, which were clinically significant [19]. Therefore, they concluded that the fortification of micronutrients, especially copper, in the Saudi diet is recommended for diabetes management [19].

A non-randomized controlled trial was performed by Armstrong et al. to assess the effect of dietary treatment for newly diagnosed adults on the complications of diabetes [28]. The twenty participants that were in the intervention group had no cardiovascular diseases and only two had a background of diabetic retinopathy compared to the twenty healthy individuals [28]. No difference was difference in copper intakes [28]. They also found that, in the cases group, fasting blood glucose levels dropped from 13.6 at the beginning of the study to 9.7 mmol/L after adhering to the diet (*p* < 0.01). HbA1c also reduced from 7.44 ± 0.67% to 5.91 ± 0.57% (*p* < 0.01) [28]. Another clinical trial performed by Rostami et al. assessed the impact of spirulina supplementation on diabetes outcomes. Each 100 g of spirulina powder contains 0.7 mg of copper. Spirulina supplementation was able to significantly decrease the total cholesterol, LDL cholesterol, triglycerides and malondialdehyde serum level significantly [27]. This indicates that spirulina may help people with diabetes to control their lipid profile [27]. However, baseline triglyceride levels were higher in the spirulina-treated diabetic patients compared to the diabetic control group [27].

## 4. Discussion

This systematic review was conducted to elucidate the studies examining the association between copper intake and the development and management of diabetes. The final review included 3 cohort studies, 4 cross-sectional studies, 2 RCT, and 2 interventional studies. The bioavailability of copper is estimated to be between 30–40% of the ingested copper amount [30]. To begin the absorption process for copper, it first needs to be reduced by several reductase enzymes from dietary cupric (Cu^2+^) to cuprous copper (Cu^1+^) [31]. Then, it is taken up across the apical membrane by copper transporter 1 (CTR1) [31]. Once copper is inside the epithelial intestinal cell, it is transferred to the protein ATOX1, and then, in order to be exported to the portal vein, it is delivered to the protein ATP7A. Any excess copper is bound to metallothionein, which is a homeostatic strategy to the existence of avoid copper toxicity inside the cytosol of the epithelial intestinal cells [31]. After copper is absorbed, 95% of it binds to ceruloplasmin in the blood [32].

Several studies have found that serum copper concentrations are higher in diabetic patients compared to healthy subjects [33,34]. In gestational diabetes, studies have found relatively high levels in pregnant women, which may be explained by the elevated copper demand as it has a vital role in embryonic and fetal growth and development [35]. Copper is one of the most important trace minerals amongst those that work as an anti-oxidant involved in redox reactions [36]. Copper is an essential element of the enzyme copper/zinc superoxide dismutase (Cu/Zn SOD) which aids in the clearance of free radicals that accumulate in cells as a result of metabolic stress [36]. Metabolic stress in an important factor that can lead to the development of comorbid conditions such as diabetes. Moreover, one mechanism explaining the association between copper and diabetes is its involvement in the reactions of glutamic acid decarboxylase (GAD), a major beta cell anti-oxidant that is altered by ROS [36]. This explanation was provided through a study investigating the association between copper and diabetes which found that the presence of anti-GAD antibodies contribute to the pathogenesis of diabetes [36]. Copper also plays an essential role in regulating ROS production through its role in the electron transfer chain, which is highly reactive in redox reactions [35]. The negative impacts of metals are frequently attributed to the generation of free radicals, including copper. Studies have suggested that copper, especially at toxic levels, may have pro-oxidant activity. Therefore, a high intake of copper elevates oxidative stress, which may contribute to an increased body inflammation [36]. Copper can cause the production of reactive oxygen species (ROS) through the Fenton reaction, which can hinder various physiological processes, including those associated with insulin resistance development and abnormal glucose metabolism [35,37]. A study conducted by Galhardi et al. showed that high copper intake promoted oxidative stress development, as well as kidney dysfunction, among rats with diabetes [16]. Another study conducted on rats found that copper deprivation, achieved through feeding them copper-deficient diet containing copper chelating agent, contributed to alpha and beta pancreatic cell neogenesis [38]. In addition, a meta-analysis conducted in 2017 reported that the risk of diabetes was positively associated with serum copper intake [39]. Moreover, a review by Kaur et al. reported that copper level abnormalities, which include both toxic and deficient levels, may induce the pathogenesis of diabetes through causing oxidative damage [40]. Previous research showed that ROS may impair insulin-dependent glucose transport by regulating the activity of several signaling molecules such as MP-activated protein kinase (AMPK), p38 mitogen-activated protein kinases (p38MAPK), and Jun *N*-terminal kinase/stress-activated protein kinase (JNK/SAPK) [36]. In addition, the activation of these signaling molecules can hinder insulin-dependent glucose transportation, resulting in insulin resistance [36]. Another mechanism involved is the potential adverse effect of copper copper on pancreatic islets [35]. It has been suggested that copper ions trigger amylin peptide aggregation to amyloid fibril, which may be related to the decreased β-cell mass along with the progressive failure of islet β-cells in human body [35].

## 5. Strengths and Limitations of the Review

This systematic review has many strengths. Up to our knowledge, this is the first systematic review to evaluate the association between copper intake and type 2 diabetes and summarize almost all the available evidence regarding this association. In addition, this review was developed based on PRISMA, and the Rayyan application was used to perform initial screening, which enabled the research team to conduct blind review that would enhance the credibility of the findings. On the other hand, there are some limitations to this systematic review. Firstly, the majority of the studies included in this review are observational, with only two randomized controlled trials, making it difficult to draw a robust conclusion from the available papers presented in this review. Furthermore, we only included papers written in English, while all other foreign languages were excluded, limiting evidence provided by this review to English literature only. Additionally, some of the studies had high risk of bias and poor overall quality, making it difficult to draw firm conclusions from them. Finally, the dietary assessment methods used in the included studies are heterogenous and have variable validity and reliability, affecting their risk of bias and the strength of the evidence they provide. It is also important to note that dietary assessment methods have limited strength compared to biochemical indicators of copper intake/status.

## 6. Conclusions

To conclude, the physiological role of copper is dependent on its level. It has a dual action, as it can work as an anti-oxidant at adequate levels and as a pro-oxidant in excessive level. Based on this review, the findings regarding the association between copper intake and the risk of diabetes are inconsistent. However, when it comes to dietary recommendations regarding copper, it is important to consider individual differences in the intake. Individuals should receive an adequate dietary amount of copper that is within the RDA levels (900 µg/day) in order to avoid copper deficiency or toxicity. It is crucial to highlight the possible toxicity of copper, which may mainly be from supplements. Supplements can be recommended among severely deficient individuals who are unable to achieve adequate copper intake from diet. Further studies, especially RCTs, are strongly needed enable researchers to elucidate the association and present a more robust conclusion.

## Figures and Tables

**Table 1:** Search Terms used in Medline, Embase, and CINAHL.

Database	Search Terms
Medline	((((((copper[MeSH Terms]) AND (Diabetes Mellitus, Type 2[MeSH Terms])) OR (Diabetes Mellitus, Non Insulin Dependent)) OR (Diabetes Mellitus, Ketosis Resistant)) OR (Diabetes Mellitus, Stable)) OR (Diabetes Mellitus, Maturity Onset)) OR (Diabetes Mellitus, Slow-Onset)
Embase	(‘copper intake’ OR ‘copper feeding’ OR ‘diet copper’ OR ‘dietary copper’ OR ‘dietary copper intake’ OR ‘copper’ OR ‘Cu^+^’ OR ‘Cu (I)’ OR ‘copper (I)’ OR ‘copper (II)’ OR ‘Cu (II)’ OR ‘Cu^++^’ OR ‘Cu^2+^’) AND (‘diabetes mellitus’ OR ‘diabetes’ OR ‘diabetic’ OR ‘non-insulin dependent diabetes mellitus’ OR ‘type 2 diabetes mellitus’)
CINAHL	(MM “copper”) AND ((MM “Diabetes Mellitus”) OR (MM “Diabetes Mellitus, Type 2”) OR (MM “Glucose Metabolism Disorders”) OR (MM “Diabetic Ketoacidosis”))

**Table 2:** Characteristics of included studies.

Study (Author, Year)	Country	Setting	Sample Size	Population and Sample Characteristics	Exposure and Outcome Assessment	Main Findings
**Randomized Controlled Trial (RCT)**						
Gunasekara et al., 2011 [18]	Sri Lanka	Patients previously diagnosed with adult-onset T2DM, for a minimum of two years, who visited the medical clinics of the teaching hospital in Karapitiya, Galle.	96	33 males, 63 females Zinc + MVM group: *n* = 29 MVM group: *n* = 31 Control group: *n* = 36 No difference in age distribution of patients in the three groups. At baseline and during the follow-up period, the three groups did not exhibit any significant differences in the use of statins and other medications that protect the heart. There were no notable differences in the dietary intake of trace minerals and vitamins.	Exposure: 3 treatment groups: Group A: zinc + MVM Group B: same MVM without zinc Group C: placebo Each MVM had 2 mg of copper included. No mention of food compsotion tables were used. Assessment: Assessed FBS and HbA1c (Mainly looked at the difference between with or without zinc)	In comparison to the other two groups (MVM and control), the zinc + MVM group had a mean change in FBS of −0.33 mmol/L (mean + SD 0.21 mmol/L; *p* = 0.05). Meanwhile, a significant decrease in HbA1C% levels was observed in zinc + MVM supplemented individuals, regardless of their baseline levels, whereas the other two groups did not exhibit any significant changes in HbA1C% levels. The MVM group showed a mean change of +0.19 mmol/L, while the control group showed a mean change of +0.43 mmol/L.
Alfawaz et al., 2019 [19]	Saudi Arabia	Al-Quds Health Care Center and specified schools located in Riyadh city	160	The GA group consisted of 75 individuals (41 males and 34 females), of which 93.3% were classified as overweight or obese. The Guidance group comprised of 64 individuals (26 males and 38 females), of which 90.6% were classified as overweight or obese. All general and clinical characteristics were not significantly different except for a slight variation in mean body mass index (BMI) (*p* = 0.04).	Group 1 (GA): provided with general information regarding the risk factors associated with prediabetes and diabetes, and a brief overview of preventive measures during the orientation. Group 2 (guidance): received comprehensive and well-organized counseling on nutrition and lifestyle related to prediabetes, diabetes, weight management, physical activity, and nutrition during their regular visits to the centers. They also attended workshops on diabetes. Outcomes: changes in glycemic indices Assessment: Took 3 24 h recalls + asked about dietary preferences. Diet was analyzed using the Food Processor Nutrition Analysis Program (ESHA).	In the GA group, a significant increase in consumption of copper was observed. Between-group comparisons revealed a clinically significant increase in copper (*p* = 0.03) in favor of the guidance group. FBG and hbA1c significantly reduced overtime in guidance group but not GA group. Insulin was reduced significantly in both groups’ overtime (*p* < 0.05 and <0.01 in GA and guidance groups, respectively) Between-group comparison showed clinically significant differences in favor of the guidance group in terms of fasting glucose (*p* = 0.005), HbA1c (*p* = 0.005), and HOMA-IR (*p* = 0.034)
**Cross-sectional Studies**						
Zhang et al., 2022 [20]	China	NHANES	4595	Nationally representative U.S population. Adults aged >62 years old. 49.79% males, 50.21% females. 978 of the 4595 participants self-reported “eye afflication/retinopathy in individuals with diabetes”. When comparing diabetic patients who reported having eye problems or retinopathy with those who did not report any eye problems or retinopathy, there were no notable differences in terms of age, sex, blood pressure, BMI, smoking, or drinking. Nevertheless, there were notable differences in duration of diabetes, insulin usage, race, and level of education.	Exposure: 58 different dietary nutrients, including copper intake (mg). Assessed through the average intake of two 24 h recalls. There was no mention of food composition tables used. Outcome: Self-reported eye affliction/retinopathy	Among the 58 dietary nutrients, fiber, butonic, octonic, vitamin A, alpha-carotene, folate, magnesium, copper and caffeine intake, reduced the occurrence of diabetic retinopathy. Logistic regression analysis showed that dietary copper intake reduces the risk of diabetic retinopathy and the pooled OR (95% CI) was 0.67 (0.54–0.84) after comparing Q4 (highest) with Q1(lowest) of the intake.
Tan et al., 2021 [21]	UK	The study recruited participants through a random selection process, utilizing advertisements and flyers that were distributed in various locations such as the University of Nottingham Malaysia and nearby supermarkets and schools.	128	The study included adult participants with an average age of 44.0 ± 10.9 years, with most of them being female (84.4%) and the rest being male (15.6%). Of the total participants, 45% (57) were found to have insulin resistance, which was defined as HOMA-IR ≥ 1.7. Participants were then separated into two groups, insulin-resistant and non-insulin-resistant, and no significant differences were observed between the two groups in terms of age, sex, race, physical activity status, smoking, or alcohol consumption.	Exposure: Dietary intake of copper and selenium. FFQ was used. Food composition was analyzed by using Dietplan7 database and “Nutrient Composition of Malaysian Foods”. Outcome: Overweight and obese participants from Malaysia and identified individuals with insulin resistance, based on a HOMA-IR value of ≥1.7.	The study found a significant positive association between dietary copper intake and HOMA-IR, but only when copper intake was ≥13.4 μg/kg/day. This association was demonstrated by an odds ratio of 0.276 (95% CI: 0.025–0.527) and a *p*-trend value of 0.033. Balance between selenium and copper intake is important, especially for individuals with diabetes and insulin resistance.
Norbitt et al., 2021 [22]	New Zealand	ANDROMEDA project	106	37 females, 69 males 3 Groups: ○ 37 New-onset diabetes or prediabetes after acute pancreatitis (NODAP) ○ 37 T2DM ○ 32 normoglycaemia after acute pancreatitis (NAP) The study found a significant statistical difference between the three groups, with the group of individuals with T2DM having the highest ratio of visceral to subcutaneous fat volume, use of antidiabetic medications, HbA1c, and FPG.	Exposure: The EPIC-Norfolk food frequency questionnaire (FFQ) was used to evaluate the typical diet of each participant throughout the year. The FETA (FFQ EPIC Tool for Analysis) software was utilized to analyze the data collected from the FFQ. Outcome: DEP Criteria NAP: HbA1c < 5.7% (39 mmol/mol) and/or FPG < 100 mg/dL (5.6 mmol/L) at initial AP and during the study. T2DM: HbA1c levels greater than or equal to 5.7% and/or FPG levels greater than or equal to 100 mg/dL before the onset of AP and at the time of the study. NODAP: Did not have hyperglycemia before and during the initial AP attack. However, during the follow-up, had HbA1c levels greater than or equal to 5.7% and/or FPG levels greater than or equal to 100 mg/dL.	The T2DM group showed a significant difference in their copper intake after taking into account their age, gender, daily energy intake, V/S fat volume ratio, alcohol consumption, and smoking status. In the NAP group, there was a significant negative correlation between FPG levels and copper intake.
Sobhani et al., 2021 [23]	Iran	Diabetes nephropathy patients randomly enrolled from Alzzahra Hospital, and a nephrology and nutrition clinic in Isfahan, Iran from July 2010 to April 2013.	397	397 patients were selected, with mean age of 65.54 ± 9.76 years, and BMI of 23.35 ± 3.53 kg/m^2^. The dependent variable used was GFR (measured in ml/min) and the presence of DN was confirmed by a nephrologist.	Exposure: Fast food consumption was measured using a validated semi-quantitative FFQ. Food composition was analyzed using NUTRITIONIST-IV software. Outcomes: Anthropometric measurements including weight, height, BMI, and waist circumference. Additionally, measurements of blood pressure, physical activity status, socioeconomic status, use of medications, biochemical analysis of lipid profile, FBS, HbA1c, BUN creatinine, and hs-CRP.	Increased consumption of fast food was linked to higher levels of energy, protein, carbohydrates, fat, cholesterol, vitamin C, saturated fat, monounsaturated fat, oleic fat, polyunsaturated fat, iron, zinc, magnesium, alpha-tocopherol, phosphorus, potassium, calcium, copper, sodium, vitamin B1, vitamin B2, vitamin B3, vitamin B6, folate, and vitamin B12. The highest intake of fast food was associated with higher levels of creatinine, systolic blood pressure, and diastolic blood pressure, and lower levels of total cholesterol.
**Cohort Studies**						
Eshak et al., 2018 [24]	Japan	Taken from 45 Japanese communities	16,160	5955 males, 10,205 females. Followed up for a period of 5 years. 396 subjects developed T2DM, 200 men and 196 women. Individuals who developed diabetes were more likely to have a family history of diabetes and hypertension compared to those who remained non-diabetic. Additionally, they were older, had a higher BMI, and were more likely to smoke and consume more alcohol (this information was not displayed in the table).	Assessment: 40-item FFQ without specifying portion sizes. Did not take into consideration copper from supplements, only diet. No mention of any food composition tables used.	The consumption of iron and copper was linked to an increased risk of developing T2DM.
Cui et al., 2022 [25]	China	Data from CHN wave 1997–2015	14,711	The study included 7333 males and 7378 females, with a mean age of 44 ± 15 years and a mean BMI of 23.2 ± 3.3 kg/m^2^. They were followed up with for 15 years. On average, participants consumed 1.9 ± 0.6 mg/day of 25Cu. Participants with higher Cu intake tended to be younger, have lower BMI, live in less urban areas and northern provinces, have lower education levels, drink alcohol, be physically active, and consume more dietary Se, total energy, protein, plant protein, carbohydrates, and fiber at a higher PUFA:SFA ratio. They also tended to consume less animal protein, have a lower animal protein to plant protein ratio, and consume less dietary fat, SFA, MUFA, and PUFA compared to those with lower Cu intake.	Assessment: The participants’ dietary intake was assessed using three consecutive 24 h recalls and was confirmed through food weighing methods at the household level. Food composition was obtained from the China Food Composition Tables.	There was no significant association between dietary intake of Cu and the risk of T2DM.
Laouali et al., 2021 [26]	France	E3N cohort, a french prospective study. Women were selected from the French national health insurance plan for teachers and coworkers, the Mutuelle Gínírale de l’Education Nationale.	70,991	The average daily intake of copper and Cu/Zn ratio was 2.90 mg (with a standard deviation of 1.15) and 0.26 (with a standard deviation of 0.10), respectively. The average age of the participants was 53 years old (with a standard deviation of 6.7), with a follow-up period of 20 years. Almost all of the women (99%) consumed more copper than the recommended amount. A higher Cu/Zn ratio was linked to older age, higher BMI and physical activity levels, and a greater incidence of hypercholesterolemia at the start of the study. Women who followed a more cautious diet had a higher Cu/Zn ratio.	Assessment: validated 208-item semi-quantitative FFQ at baseline in 1993. A follow-up questionnaire was sent to identify potential T2DM cases. A food composition database adapted from the French Information Center on Food Quality was used.	A reduced Cu/Zn ratio in one’s diet is linked to a decreased risk of developing Type 2 Diabetes, particularly in women who are obese and consume more than 8 mg of zinc per day.
**Non-randomized controlled trials**						
Rostami et al., 2022 [27]	Iran	New cases of types 2 diabetes referred to the health center in Behshar, Mazandaran Province, Iran.	30	15 individuals did not consume any functional foods or supplements, and were not given spirulina supplements. Another 15 individuals did not consume any oral hypoglycemic medications, insulin, functional foods, or other supplements, but did receive spirulina supplements. Participants consisted of 11 males and 19 females. Mean ages for spirulina group and controls were 46.70 ± 8.10 and 47.30 ± 8.80, respectively. The BMI of the group receiving spirulina treatment was 28.27 (with a standard deviation of 2.05), while the BMI of the diabetic control group was 27.21 (with a standard deviation of 1.83).	Exposure: Spirulina supplementation as pills were given for eight weeks (4 g/day, which contains 280 mcg of copper). No mention of food composition tables used. Outcomes: 12 h overnight fasting blood glucose, triglyceride, cholesterol, HDL cholesterol, LDL cholesterol, and MDA, serum insulin, in addition to weight, height, and BMI.	There was a noticeable reduction in the levels of total cholesterol, LDL cholesterol, triglycerides, and MDA in the blood serum. Upon analysis of the correlation, it was found that higher levels of serum triglycerides, total cholesterol, LDL cholesterol, and MDA at the start of the study were linked to a greater decrease in the lipid profile and MDA levels. The serum MDA levels of diabetic participants exhibited a significant decrease after receiving spirulina supplements, dropping from 6.0 nm/L (with a standard deviation of 1.43) to 4.88 nm/L (with a standard deviation of 1.16).
Armstrong et al., 1995 [28]	Ireland	Cases: from diabetes clinic Controls: hospital staff and other outpatient clinics	40 (20 cases, 20 controls)	10 males and 10 females in each group, each group had 6 smokers. Average age for control was 54 years, and average age for cases was 58 years. Average BMI for control was 26, and average BMI for cases was 31.	Exposure: The individuals in the experimental group were provided with dietary guidance based on the 1990 revision of the dietary recommendations for individuals with diabetes. The control group received comparable treatment to the experimental group, with the exception that no dietary advice was offered to them. Assessment: One 24 h recall, FFQ, fasting glucose. Diet was analyzed using MICRO-DIET computer software based on McCance and Widdowson’s Composition of Food.	The intake of copper was comparable in both the diabetic patients and the control group, but the exact amounts were not specified. In diabetic patients, the fasting blood glucose (FBG) levels decreased from 13.6 mmol/L at the beginning of the study to 9.7 mmol/L after dietary intervention. HbA1c reduced from 7.44 ± 0.67% to 5.91 ± 0.57% (*p* < 0.01).

**Table 3:** Summary of the quality assessment of studies using Newcastle–Ottawa Scale.

Cross-Sectional Studies					
Study	Selection	Comparability	Outcome	Total Quality Score	
Zhang et al., 2022 [20]	** (Representative of target population; unsatisfactory response rate; validated measurement tool)	** (Multiple confounders are controlled)	* (Self-reported outcomes; clear statistical tool)	5	
Tan et al., 2021 [21]	** (Selection of users was volunteer-based, but somewhat representative of target population; poor description of response rate; validated measurement tools were used	** (Multiple confounders are controlled)	** Self-reported outcomes with record linkages; clear statistical tool)	6	
Norbitt, Kimita, Ko, Bharmal, and Petrov, 2021 [22]	*** (Selection was representative of population; satisfactory response rate; validated measurement tool)	* (Important confounders are controlled)	** (Self-reported outcomes with record linkages; appropriate statistical methods)	6	
Sobhani et al., 2021 [23]	*** (Selected users; sample size calculations were shown; non-respondents were not mentioned in the study; exposure: “DN was affirmed by a nephrologist”)	** (Author controlled for multiple confounders)	** (Validated FFQ was used; statistical method not appropriate, no association was tested, and author only reported mean values at each tertile of fast food intake)	5	
**Cohort Studies**					
**Study**	**Selection**	**Comparability**	**Outcome**	**Total Quality Score**	**Comment**
Eshak et al., 2018 [24]	**** (Representative of target population; selection from same community as exposed cohort; structured interviews given; demonstrated that outcome was not present at start of study)	** (Authors controlled for important and additional confounders)	* (Self-reported outcomes; follow-up period of 5 years was inadequate; adequate subjects accounted for)	7	
Cui et al., 2022 [25]	**** (Representative of target population; selection from same community as exposed cohort; structured interviews given and records reviewed; demonstrated that outcome was not present at start of study)	** (Authors controlled for important and additional confounders)	*** (Outcomes are linked with records in addition to self-reported outcomes; adequate subjects accounted for; adequate follow-up period of 15 years)	9	
Laouali et al., 2021 [26]	*** (Representative of target population; selection from same community as exposed cohort; structured interviews given and records reviewed; demonstrated that outcome was not present at start of study)	* (Authors controlled for important confounders)	** (Self-reported outcomes; adequate subjects accounted for; adequate follow-up period of 20 years)	6	In the selection; ascertainment of exposure was self-reported

Note: Each asterisk represents a point awarded for each section and serves as a quick visual assessment of the quality of the study. Possible total points for each section are 4 for Selection, 2 for Comparability, and 3 for Outcome.

**Table 4:** Summary of the quality assessment of studies using Cochrane risk-of-bias tool for RCT’s.

RCT Studies								
Study	Domain 1 Risk of bias arising from the randomization process	Domain 2 Risk of bias due to deviations from the intended interventions (effect of assignment/adhering to intervention)	Domain 3 Missing outcome data	Domain 4 Risk of bias in measurement of the outcome	Domain 5 Risk of bias in selection of the reported result			Overall risk of bias
Gunasekara et al., 2011 [18] (RCT)	Some concerns Difference between groups not fully described, which may suggest a problem with randomization	Low (effect assignment to intervention) Some concerns (effect of adhering to intervention) due complain to medication inquired/single-blinded	Some concerns out of 96, only 86 completed the study (risk of missing data in the outcome depend on its true value)	Low	Low			Low risk of bias
Alfawaz et al., 2019 [19] (RCT)	High Due to baseline differences between groups	High (effect assignment to intervention) Participants and people delivering the intervention were aware of their intervention. Some concerns (effect of adhering to intervention) due to compliance to dietary recommendation	Low	High Assessment of the outcome could have been influenced by knowledge of intervention receive	Low			High risk of bias
Non-RCT studies	Domain 1 Bias due to confounding	Domain 2 Bias in selection of participants into the study	Domain 3 Bias in classification of interventions	Domain 4 Bias due to deviations from intended interventions	Domain 5 Bias due to missing data	Domain 6 Bias in measurement of outcomes	Domain 7 Bias in selection of the reported result	Overall risk of bias
Rostami et al., 2022 [27] (non-RCT)	Modetate	Low	Low	Low	Low	Moderate	Moderate	Moderate
Armstrong et al., 1995 [28] (non-RCT)	Moderate	Serious	Moderate	Moderate	Moderate	Moderate	Serious	Serious

## Data Availability

Not applicable.

## Supplementary Materials

The following supporting information can be downloaded at: https://www.mdpi.com/article/10.3390/nu15071655/s1, Table S1: PRISMA 2020 Checklist. Reference [40] is cited in the Supplementary Materials.

## References

[1] Sapra A., Bhandari P., Hughes A.W. (2021). Diabetes Mellitus (Nursing).

[2] Al-Lawati J.A. (2017). Diabetes mellitus: A local and global public health emergency!. Oman Med. J..

[3] Diabetes. https://www.who.int/news-room/fact-sheets/detail/diabetes.

[4] Lam A.A., Lepe A., Wild S.H., Jackson C. (2021). Diabetes comorbidities in low-and middle-income countries: An umbrella review. J. Glob. Health.

[5] Pham T.B., Nguyen T.T., Truong H.T., Trinh C.H., Du H.N.T., Ngo T.T., Nguyen L.H. (2020). Effects of diabetic complications on health-related quality of life impairment in Vietnamese patients with type 2 diabetes. J. Diabetes Res..

[6] Diabetes Symptoms|CDC. https://www.cdc.gov/diabetes/basics/symptoms.html.

[7] Yoon J.-W., Jun H.-S. (2005). Autoimmune destruction of pancreatic β cells. Am. J. Ther..

[8] Murea M., Ma L., Freedman B.I. (2012). Genetic and environmental factors associated with type 2 diabetes and diabetic vascular complications. Rev. Diabet. Stud. RDS.

[9] Symptoms & Causes of Diabetes|NIDDK. https://www.niddk.nih.gov/health-information/diabetes/overview/symptoms-causes.

[10] Diabetes|Division of Global Health Protection|Global Health|CDC. https://www.cdc.gov/globalhealth/healthprotection/ncd/diabetes.html.

[11] Mekhilef S., Saidur R., Kamalisarvestani M. (2012). Effect of dust, humidity and air velocity on efficiency of photovoltaic cells. Renew. Sustain. Energy Rev..

[12] Bost M., Houdart S., Oberli M., Kalonji E., Huneau J.F., Margaritis I. (2016). Dietary copper and human health: Current evidence and unresolved issues. J. Trace Elem. Med. Biol. Organ Soc. Miner. Trace Elem. (GMS).

[13] Yin J.-J., Fu P.P., Lutterodt H., Zhou Y.-T., Antholine W.E., Wamer W. (2012). Dual role of selected antioxidants found in dietary supplements: Crossover between anti-and pro-oxidant activities in the presence of copper. J. Agric. Food Chem..

[14] Maritim A.C., Sanders R.A., Watkins J.B. (2003). Diabetes, oxidative stress, and antioxidants: A review. J. Biochem. Mol. Toxicol..

[15] Fujii J., Homma T., Osaki T. (2022). Superoxide Radicals in the Execution of Cell Death. Antioxidants.

[16] Galhardi C.M., Diniz Y.S., Faine L.A., Rodrigues H.G., Burneiko R.C.M., Ribas B.O., Novelli E.L. (2004). Toxicity of copper intake: Lipid profile, oxidative stress and susceptibility to renal dysfunction. Food Chem. Toxicol..

[17] Jomova K., Valko M. (2011). Advances in metal-induced oxidative stress and human disease. Toxicology.

[18] Hettiarachchi M., Gunasekara P., Liyanage C., Lekamwasam S. (2011). Effects of zinc and multimineral vitamin supplementation on glycemic and lipid control in adult diabetes. Diabetes Metab. Syndr. Obes. Targets Ther..

[19] Alfawaz H., Naeef A.F., Wani K., Khattak M.N.K., Sabico S., Alnaami A.M., Al-Daghri N.M. (2019). Improvements in glycemic, micronutrient, and mineral indices in Arab adults with pre-diabetes post-lifestyle modification program. Nutrients.

[20] Zhang G., Sun X., Yuan T., Guo C., Zhou Z., Wang L., Dou G. (2022). Certain Dietary Nutrients Reduce the Risk of Eye Affliction/Retinopathy in Individuals with Diabetes: National Health and Nutrition Examination Survey, 2003–2018. Int. J. Environ. Res. Public Health.

[21] Tan P.Y., Mitra S.R. (2021). Dietary copper and selenium are associated with insulin resistance in overweight and obese Malaysian adults. Nutr. Res..

[22] Norbitt C.F., Kimita W., Ko J., Bharmal S.H., Petrov M.S. (2021). Associations of habitual mineral intake with new-onset prediabetes/diabetes after acute pancreatitis. Nutrients.

[23] Sobhani S.R., Mortazavi M., Kazemifar M., Azadbakht L. (2021). The association between fast-food consumption with cardiovascular diseases risk factors and kidney function in patients with diabetic nephropathy. J. Cardiovasc. Thorac. Res..

[24] Eshak E.S., Iso H., Maruyama K., Muraki I., Tamakoshi A. (2018). Associations between dietary intakes of iron, copper and zinc with risk of type 2 diabetes mellitus: A large population-based prospective cohort study. Clin. Nutr..

[25] Cui Z., Zhou H., Liu K., Wu M., Li S., Meng S., Meng H. (2022). Dietary Copper and Selenium Intakes and the Risk of Type 2 Diabetes Mellitus: Findings from the China Health and Nutrition Survey. Nutrients.

[26] Laouali N., MacDonald C.-J., Shah S., El Fatouhi D., Mancini F., Fagherazzi G., Boutron-Ruault M.-C. (2021). Dietary copper/zinc ratio and type 2 diabetes risk in women: The E3N cohort study. Nutrients.

[27] Rostami H.A.A., Marjani A., Mojerloo M., Rahimi B., Marjani M. (2022). Effect of Spirulina on Lipid Profile, Glucose and Malondialdehyde Levels in Type 2 Diabetic Patients. Braz. J. Pharm. Sci..

[28] Armstrong A.M., Chestnutt J.E., Gormley M.J., Young I.S. (1996). The effect of dietary treatment on lipid peroxidation and antioxidant status in newly diagnosed noninsulin dependent diabetes. Free Radic. Biol. Med..

[29] Newcastle-Ottawa Scale. https://www.sciencedirect.com/topics/nursing-and-health-professions/newcastle-ottawa-scale.

[30] Wapnir R.A. (1998). Copper absorption and bioavailability. Am. J. Clin. Nutr..

[31] Nishito Y., Kambe T. (2018). Absorption mechanisms of iron, copper, and zinc: An overview. J. Nutr. Sci. Vitaminol..

[32] Hellman N.E., Kono S., Mancini G.M., Hoogeboom A.J., de Jong G.J., Gitlin J.D. (2002). Mechanisms of copper incorporation into human ceruloplasmin. J. Biol. Chem..

[33] Lin C.-C., Huang H.-H., Hu C.-W., Chen B.-H., Chong I.-W., Chao Y.-Y., Huang Y.-L. (2014). Trace elements, oxidative stress and glycemic control in young people with type 1 diabetes mellitus. J. Trace Elem. Med. Biol..

[34] Naka T., Kaneto H., Katakami N., Matsuoka T.-A., Harada A., Yamasaki Y., Matsuhisa M., Shimomura I. (2013). Association of serum copper levels and glycemic control in patients with type 2 diabetes. Endocr. J..

[35] Lian S., Zhang T., Yu Y., Zhang B. (2021). Relationship of circulating copper level with gestational diabetes mellitus: A meta-analysis and systemic review. Biol. Trace Elem. Res..

[36] Miyazaki T., Takenaka T., Inoue T., Sato M., Miyajima Y., Nodera M., Hanyu M., Ohno Y., Shibazaki S., Suzuki H. (2012). Lipopolysaccharide-induced overproduction of nitric oxide and overexpression of iNOS and interleukin-1β proteins in zinc-deficient rats. Biol. Trace Elem. Res..

[37] Basu A., Alman A.C., Snell-Bergeon J.K. (2022). Associations of Dietary Antioxidants with Glycated Hemoglobin and Insulin Sensitivity in Adults with and without Type 1 Diabetes. J. Diabetes Res..

[38] Al-Abdullah I.H., Ayala G., Panigrahi D., Kumar A.M.S., Kumar M.S.A. (2000). Neogenesis of pancreatic endocrine cells in copper-deprived rat models. Pancreas.

[39] Qiu Q., Zhang F., Zhu W., Wu J., Liang M. (2017). Copper in diabetes mellitus: A meta-analysis and systematic review of plasma and serum studies. Biol. Trace Elem. Res..

[40] Page M.J., McKenzie J.E., Bossuyt P.M., Boutron I., Hoffmann T.C., Mulrow C.D., Shamseer L., Tetzlaff J.M., Akl E.A., Brennan S.E. (2021). The PRISMA 2020 statement: An updated guideline for reporting systematic reviews. BMJ.

